# Susceptibility of optic nerve head in children with posture-related elevation of intraocular pressure

**DOI:** 10.1007/s10792-024-03109-6

**Published:** 2024-04-16

**Authors:** Jan Niklas Lüke, Caroline Gietzelt, Philip Enders, Johanna Dietlein, Alexandra Lappa, Vincent Lüke, Randolf Alexander Widder, Thomas S. Dietlein

**Affiliations:** 1 Department of Ophthalmology, Medical Faculty and University Hospital of Cologne, Kerpener Strasse 62, 50937 Cologne, Germany; 2 Department of Ophthalmology, St. Martinus-Krankenhaus Düsseldorf, Gladbacher Str. 26, 40219 Düsseldorf, Germany

**Keywords:** Bruch membrane opening, Intraocular pressure, Glaucoma, Posture-related, Children, Rebound tonometry

## Abstract

**Background/Aims:**

This work aimed to investigate changes in optic nerve head (ONH) morphometry based on Bruch membrane opening in children with extensive nocturnal intraocular pressure (IOP) elevations.

**Methods:**

The course of Bruch membrane opening-based optic nerve head (ONH) morphometry was analysed in thirty-two patients younger than 18 years with evaluable SD-OCT examinations of the ONH and nocturnal posture-dependent IOP elevation above 25 mmHg. Longitudinal changes in neuroretinal rim tissue, as measured by Bruch Membrane opening minimum rim width (BMO-MRW) and peripapillary retinal nerve fiber layer (RNFL) thickness, were assessed.

**Results:**

One year after the 24 h IOP measurement, global BMO-MRW (− 1.61 ± 16.8 µm, n.s.; *p* = 0.611) and RNFL (+ 0.64 ± 3.17 µm; n.s.; *p* = 0.292) measurements were not significantly different from the baseline. No significant BMO-MRW reduction (− 3.91 ± 24.3 µm; n.s. *p* = 0.458) or deviation in RNFL thickness (+ 1.10 ± 3.52 µm) was observed at the four-year follow-up. Absolute IOP values measured in the supine position did not correlate with changes in global BMO-MRW or RNFL thickness.

**Conclusion:**

Posture-dependent IOP elevations do not seem to influence retinal nerve fibre layer thickness or Bruch membrane opening-based morphometric data in childhood.

## Introduction

The phenomenon of diurnal, pronounced nocturnal, and positional fluctuations of intraocular pressure (IOP) in adults has been the subject of several studies. A clustered occurrence of these fluctuations, as well as an increased range of fluctuation in glaucoma patients, is assumed [1]. In addition, patient age appears to influence the likelihood of a greater nocturnal IOP increase [1]. The development of postural IOP spikes seems to have multifactorial causes but is probably mainly due to increased episcleral venous pressure.

Several studies have been initiated to determine the magnitude of IOP fluctuations in adult patients. Gautam et al. measured a postural fluctuation of 4.5 ± 1.24 mmHg in adult patients diagnosed with glaucoma and 2.2 ± 1.1 mmHg in healthy subjects. Mean daily fluctuations were described as 5.67 mmHg ± 5.11 mmHg (POAG) and 3.1 ± 2.9 mmHg (healthy subjects) [2].

Currently, there is no data on 24-h IOP fluctuations in healthy individuals younger than 18 years. Most studies exclude subjects younger than 18 years. A significant nyctohemeral IOP rhythm when measuring hourly could be detected in 83% of six young healthy subjects with a mean age of 24 years [3].

A higher difference in IOP, comparing sitting with supine position measurements, was found in younger subjects (20–30 years), compared to groups older than 30 years [4].

Values for IOP measurements of young children with or without glaucoma are frequently taken under general anaesthesia. In a range between 6 and 50 mmHg, IOP in the eyes of supine children under anaesthesia measured approximately 2 mmHg higher by Tono-Pen XL than iCare Pro with greater differences in eyes with corneal edema [5]. Furthermore, it could be shown in children, that changing the body position from sitting to supine position leads to a significant elevation of the IOP 5 min later of approximately 1 mmHg [6].

The extent to which IOP variability, including short- and long-term IOP fluctuations, influences glaucoma progression in adults remains controversial and unclear [7]. In all these reviewed studies, visual field analysis was used to assess progression. In contrast, in young children, visual field analysis often cannot be considered a reliable diagnostic tool to assess disease progression. Consequently, there is a lack of information on the extent to which IOP peaks in children promote glaucoma progression.

We, therefore, attempt to approach this question by analysing Bruch membrane opening-based morphometry of children with detected IOP peaks > 25 mmHg in the 24 h measurement.

## Materials and methods

### Study design

Patients were referred to a tertiary glaucoma centre between 2015 and 2019 for measurements of diurnal and nocturnal intraocular pressure due to suspicion of glaucoma and followed up, for up to four years, using spectral-domain optical coherence tomography (SD-OCT).

Medical data of 7000 patients was screened for the following inclusion and exclusion criteria:

Only the right eye of these thirty-two patients younger than 18 years, who had values above 25 mmHg on supine intraocular pressure measurement, was included in the study. All non-glaucomatous ocular diseases that could affect the accuracy of tonometry or optic disc morphology (e.g., superficial keratopathy or prematurity) were exclusion criteria, as were syndromal diseases, high refractive error (more than + 5/ − 5dpt hyperopia or myopia), high astigmatism (more than − 3dpt), refractive surgery, or any intraocular surgery. Due to the young age of the patients, reliable visual field analysis was not available in many cases. When perimetry could be performed, no significant and reproducible defects could be detected. Perimetry was available in n = 21 cases, and reliable visual field examinations were not available in n = 11 patients.

Due to the retrospective approach, the availability of multiple follow-up examinations per patient was limited. To better analyze and investigate the follow-ups, we aggregated all patients to the following groups.

SD-OCT of ONH was performed at least at the time of the 24-h measurement and one year later (399 days ± 3 month) and/or four years later (1409 days ± 6 month). Not every patient received a follow-up SD-OCT examination after one year as well as after 4 years, some patients only at one of the two follow-up intervals. All patients who did not have OCT at the time of the 24-h measurement were excluded.

Medical data, including visual acuity, corneal thickness, topical medication, and highest measured IOP, was obtained from the medical records. IOP values were measured by rebound tonometry (Icare Tonometer TA01i, Icare Finland Oy, Vantaa, Finland). SD-OCT examinations were performed according to the standard operating procedures of the Eye Clinic of the University of Cologne [8]. Twenty-four scans with 48 measurement points of BMO-MRW and three circular scans of RNFL thickness were calculated by the Spectralis®-SD-OCT platform (Heidelberg Engineering GmbH, Heidelberg, Germany), and the export tool was used for data acquisition. Patients with unsatisfactory image quality and uncorrectable segmentation errors were excluded. When possible, segmentation errors were corrected manually.

### Ethical approval and statistical analysis

According to regulations of the professional code for Physicians, all tenets of the Declaration of Helsinki have been regarded.

In accordance with the provisions of the Code of Professional Conduct for Physicians, all principles of the Declaration of Helsinki were followed.

Descriptive statistics were performed for a more detailed characterization of the study group. Normal distribution was tested with the D’Agostino-Pearson normality test. Because the RNFL and BMO-MRW values were not normally distributed, the Wilcoxon test was performed to assess significance. In the case of normal distribution, the paired t-test was performed. Values were expressed as mean ± standard deviation of the mean (SD). Statistical significance was set at *p* < 0.05. Other high significance levels were set at *p* < 0.01 and *p* < 0.001. All analyses and data presentations were performed using Excel (Microsoft Office Excel 2016, California, USA), SPSS v. 22 (IBM Chicago, Illinois, USA), and GraphPad software (GraphPad Prism 7, Inc, La Jolla, USA).

Because not each of the 32 patients was examined at each of the time points (baseline; one year; four years), baseline BMO-MRW and mean RNFL thickness data differed for the one-year and four-year follow-up intervals, according to analyses of previous studies in our clinic [8, 9].

## Results

Thirty-two patients were included. N = 29 could be followed up for one year, and n = 22 SD-OCT examinations were evaluated after four years. The epidemiological data of our cohort can be found in Table 1.

**Table 1:** Epidemiological data of the study cohort

	Study eyes(n = 32)
*Age (years)*	
Mean ± SD	12.2 ± 3.6
Range	4–17
*BCVA at baseline in logMAR*	
Mean ± SD	0.0 ± 0.1
Range	0–0.1
*Cup-disc-Ratio*	
Mean ± SD	0.5 ± 0.1
Range	0.3–0.8
*Pachymetry (in µm)*	
Mean ± SD	580.8 ± 33.8
Range	530–624
*OCT parameters*	
Mean Global BMO-MRW in µm	296.7 ± 45.9
Mean BMO Area in mm^2^	2.2 ± 0.4
Mean Global RNFL-Thickness	94.5 ± 12.1

BCVA: Best corrected visual acuity; SD: standard deviation; BMO-MRW: Bruch Membran opening Minimum Rim Width; RNFL: Retinal nerve fibre layer; OCT: Optical coherence tomography

During 24-h IOP measurement, daily values averaged 20.8 ± 6.6 mmHg, daily fluctuation was 8.0 ± 4.6 mmHg, and supine measurements were elevated to 32.5 ± 6.5 (range 25–45 mmHg). The mean deviation of perimetry in n = 21 patients averaged − 0.2 dB ± 1.6 dB and reliable perimetry was not available in n = 11 cases.

A baseline OCT examination was performed during hospitalization for 24-h measurement of IOP. The 1-year examination was performed after 399 days ± 3 month, and the 4-year examination was performed after an average of 1409 days ± 6 month.

After one year, global BMO-MRW was not significantly different from the baseline (− 1.61 ± 16.8 µm, n.s.; *p* = 0.611). RNFL thickness also showed stable values during this period (+ 0.64 ± 3.17 µm; n.s.; *p* = 0.292). Detailed analysis of the individual sectors (temporal, temporal-inferior; temporal-superior; nasal; nasal-superior, nasal-inferior) also revealed no significant deviation from the baseline (Table 2).

**Table 2:** Mean of differences of BMO-MRW presented in µm

Baseline	G	TI	T	TS	N	NS	NI
1y:G	− 1.6 ± 16.8						
1y:TI		− 3.8 ± 28.1					
1y: T			− 3.1 ± 17.2				
1y: TS				− 0.2 ± 16.1			
1y: N					Median: + 1.13 (Wilcoxon)		
1y: NS						Median: + 0.39 (Wilcoxon)	
1y: NI							Median: + 5.7 (Wilcoxon)
4y: G	− 3.9 ± 24.3						
4y: TI		− 3.1 ± 30.8					
4y: T			+ 4.3 ± 18.0				
4y: TS				+ 5.4 ± 23.4			
4y: N					Median: − 5.4 (Wilcoxon)		
4y: NS						Median: − 1.7 (Wilcoxon)	
4y: NI							− 1.4 ± 17.3

1y = one year follow up; 4y = four years follow up, No value deviated significantly from the respective baseline value performing two-tailed paired t-test or Wilcoxon-test respectively

G:global; TI:temporal inferior; T:temporal; TS:temporal superior; n:nasal; NS:nasal superior; NI:nasal inferior; BMO-MRW: Bruch Membran opening-Minimum Rim Width

Similarly, no significant BMO-MRW reduction (− 3.91 ± 24.3 µm; n.s. *p* = 0.458) or deviation in RNFL thickness (+ 1.10 ± 3.52 µm, n.s.) was observed at the 4-year follow-up.

For the assessment of possible glaucomatous damage due to extended nocturnal IOP peaks, we considered children with values of 35 mmHg and higher. Also in this group no significant BMO-MRW reduction (1y (n = 10): + 4.44 ± 10.0 µm n.s.; *p* = 0.194; 4y (n = 8): + 6.9 ± 13.75 µm; n. s.; *p* = 0.196) or RNFL thickness reduction (1y (n = 9): + 1.22 ± 1.72 µm; n.s.; *p* = 0.065; 4y (n = 7): + 0.29 ± 1.60 µm; n.s.; *p* = 0.654) was found. (Table 3).

**Table 3:** Mean of differences of RNFL thickness in µm in the several sectors

Baseline	G	TI	T	TS	N	NS	NI
1y:G	+ 0.6 ± 3.2						
1y:TI		+ 1.5 ± 5.3					
1y: T			+ 0.6 ± 2.5				
1y: TS				+ 0.3 ± 4.2			
1y: N					+ 0.36 ± 8.9		
1y: NS						0 + .9 ± 3.1	
1y: NI							+ 0.8 ± 3.8
4y: G	+ 1.1 ± 3.5						
4y: TI		− 1.4 ± 10.1					
4y: T			+ 1.7 ± 6.3				
4y: TS				− 4.9 ± 15.7			
4y: N					+ 1 (Median, Wilcoxon)		
4y: NS						− 1.8 ± 13.1	
4y: NI							+ 1.3 ± 8.4

No value deviated significantly from the respective baseline value performing two-tailed paired t-test or Wilcoxon-test respectively

1y = one year follow-up; 4y = four years follow-up; G:global; TI:temporal inferior; T:temporal; TS:temporal superior; n:nasal; NS:nasal superior; NI:nasal inferior. RNFL: Retinal nerve fibre layer

Furthermore, no significant correlation was found between absolute values of nocturnal IOP peaks and changes in BMO-MRW (1y: Spearman’s rho = 0.176; *p* = 0.360; 4y: Spearman’s rho = 0.343; *p* = 0.118) and RNFL thickness (1y: Spearman’s rho = 0.171; *p* = 0.385; 4y: Spearman’s rho = 0.155; *p* = 0.490) over the periods.

## Discussion

In adults, diurnal and nocturnal short-term fluctuations of IOP are suggested to be associated with glaucoma progression as measured by visual field analysis [10]. In addition, there is evidence that eyes with a higher magnitude of postural change-induced IOP elevation have lower mean RNFL thickness in adult patients with primary open-angle glaucoma [11].

There is a lack of studies providing further information on the extent to which glaucoma progression is enhanced by extensive IOP variations during the day or night in childhood.

It should be noted that a progression analysis in children is much more challenging since reliable perimetry is not feasible until a certain age is reached. During this time morphometric examinations of the ONH may provide better objectifiable information. Furthermore, the focus should be on early detection of RNFL reductions in glaucoma-suspected patients at a young age, if possible, before a visual field defect occurs.

The underlying results of this study can confirm that neuroretinal tissue of the ONH was stable and not significantly reduced for four years, despite increased posture-related IOP fluctuations in history. No significant correlation between the height of the IOP peaks and the morphometric dynamics was found indicating that even IOP peaks up to 45 mmHg do not necessarily lead to glaucomatous damage in childhood.

It can be suggested that neuronal susceptibility is decreased in childhood compared to adult glaucoma patients. Some studies postulate an increased prevalence of ONH cupping reversal in younger age, which is attributed to the elastic properties of their neuronal tissues [12, 13]. In foetal and neonatal congenital glaucoma eyes, histochemical examinations indicated an incomplete collagenous structural framework of the lamina cribrosa [13].

It is discussed, controversially, whether the translamina cribrosa pressure difference (TLCPD) between the intracranial and intraocular pressure plays a role in glaucoma development, especially for normal-tension glaucoma. Some hypotheses provide an explanation for the differential susceptibility of ONH to elevated IOP:

In the supine position, retrolaminary pressure is equal to that in the optic nerve subarachnoidal space (ONSAS). In the upright position and when the intracranial pressure (ICP) drops sharply, an occlusion mechanism of the optic nerve sheath could compensate for higher pressure in the ONSAS, as it corresponds to the orbital pressure and thus keeps the TLCPD stable [14]. If this occlusion mechanism is deficient and an open fluid communication is present, which is hypothesized in normal-tension glaucoma, then retrolaminar pressure would be the same as the ICP, resulting in an elevated and potentially harmful TLCPD in an upright position [14]. Therefore, a measurement of ICP in the supine position in the children studied here would be very interesting additional, although very inaccessible information for further understanding of pathophysiology.

Mean daily intraocular pressure values have been shown to be elevated in healthy children aged nine to 11 years compared with adults [15].

Consequently, the decision to intervene due to posture-related pressure peaks should be made as strictly as possible. Nevertheless, it is essential to examine these patients systematically and regularly. Treatment options include local treatment, although poor controllability of nocturnal IOP peaks has been described in adult patients [16]. Surgical procedures, such as trabeculectomy, are thought to adequately treat IOP peaks sufficiently because they bypass the venous resistance of the episcleral veins by draining aqueous humour into the subconjunctival space. If indicated, filtration surgery such as trabeculectomy is an established procedure also in childhood [17].

Several limitations must be considered when contextualising our findings. The retrospective nature and the limited number of cases are attributable to the strict inclusion criteria and the infrequent hospitalization for 24-h measurement of children with suspected glaucoma and peak IOP values > 25 mmHg leading to a lack of information. For this reason, a longitudinal prospective study design was not feasible for this question.

It should also be noted that this study does not provide any information on the extent to which stronger IOP fluctuations lead to RNFL damage in children with clearly diagnosed glaucoma.

In addition, OCT-based analysis has several potential sources of error. In a few isolated cases, segmentation errors occurred during OCT examinations that had to be corrected manually. It can be discussed whether this leads to a similarly accurate result as automatic segmentation when no errors are present. In addition, RNFL thickness may be biased by scan circle displacement [18]. It is well known that BMO-MRW can be affected by major IOP fluctuations[19]. Thus, because the present patient cohort is known to have a high intraindividual variation in IOP, it can be assumed that BMO-MRW is also subject to increased IOP-dependent variation, which could be an explanation for the higher standard deviation values of BMO-MRW differences. In contrast, smaller intraday variations in IOP were shown not to result in significant changes in BMO-MRW or RNFL thickness [20].

In summary, our findings indicate a low level of susceptibility of the juvenile ONH against posture-dependent IOP fluctuations in a mid-term interval. There is still no information as to what extent stronger IOP fluctuations in childhood contribute to the development of glaucoma in a long-term interval. In SD-OCT examinations, neither BMO-MRW nor RNFL thickness altered significantly up to four years after measured posture-dependent IOP fluctuations.
